# Dynamic Interference Testing—Unexpected Results Obtained with the Abbott Libre 2 and Dexcom G6 Continuous Glucose Monitoring Devices †

**DOI:** 10.3390/s25071985

**Published:** 2025-03-22

**Authors:** Hendrick Jensch, Steven Setford, Nicole Thomé, Geethan Srikanthamoorthy, Lea Weingärtner, Mike Grady, Elizabeth Holt, Andreas Pfützner

**Affiliations:** 1 Pfützner Science & Health Institute, Haifa-Allee 20, 55128 Mainz, Germany; hendrick.jensch@lifecare.no (H.J.); nicole.thome@lifecare.no (N.T.); geethan.srikanthamoorthy@lifecare.no (G.S.); lea.weingaertner@lifecare.no (L.W.); 2 Lifecare Laboratories, 55128 Mainz, Germany; 3 LifeScan Scotland Ltd., Inverness IV2 2ED, UK; ssetford@lifescan.com (S.S.); mgrady@lifescan.com (M.G.); 4 LifeScan Global Corp., Malvern, PA 19355, USA; eholt@lifescan.com; 5 Department of Biotechnology, Technical University Bingen, 55411 Bingen am Rhein, Germany; 6 Institute of Internal Medicine and Laboratory Medicine, University for Digital Technologies in Medicine and Dentistry, L-9516 Wiltz, Luxembourg

**Keywords:** continuous glucose monitoring, Abbott Libre 2, dynamic interference testing, Dexcom G6, interferents

## Abstract

Background: Sensors for continuous glucose monitoring (CGM) are now commonly used by people with type 1 and type 2 diabetes. However, the response of these devices to potentially interfering nutritional, pharmaceutical, or endogenous substances is barely explored. We previously developed an in vitro test method for continuous and dynamic CGM interference testing and herein explore the sensitivity of the Abbott Libre2 (L2) and Dexcom G6 (G6) sensors to a panel of 68 individual substances. Methods: In each interference experiment, L2 and G6 sensors were exposed in triplicate to substance gradients from zero to supraphysiological concentrations at a stable glucose concentration of 200 mg/dL. YSI Stat 2300 Plus was used as the glucose reference method. Interference was presumed if the CGM sensors showed a mean bias of at least ±10% from baseline with a tested substance at any given substance concentration. Results: Both L2 and G6 sensors showed interference with the following substances: dithiothreitol (maximal bias from baseline: L2/G6: +46%/−18%), galactose (>+100%/+17%), mannose (>+100%/+20%), and N-acetyl-cysteine (+11%/+18%). The following substances were found to interfere with L2 sensors only: ascorbic acid (+48%), ibuprofen (+14%), icodextrin (+10%), methyldopa (+16%), red wine (+12%), and xylose (>+100%). On the other hand, the following substances were found to interfere with G6 sensors only: acetaminophen (>+100%), ethyl alcohol (+12%), gentisic acid (+18%), hydroxyurea (>+100%), l-cysteine (−25%), l-Dopa (+11%), and uric acid (+33%). Additionally, G6 sensors could subsequently not be calibrated for use after exposure to dithiothreitol, gentisic acid, l-cysteine, and mesalazine (sensor fouling). Conclusions: Our standardized dynamic interference testing protocol identified several nutritional, pharmaceutical and endogenous substances that substantially influenced L2 and G6 sensor signals. Clinical trials are now necessary to investigate whether our findings are of relevance during routine care.

## 1. Introduction

Minimally invasive sensors for continuous glucose monitoring (CGM) are becoming increasingly popular amongst people with type 1 and type 2 diabetes. Their application in daily practice has introduced the concept of “time in range” as a new efficacy parameter for diabetes care [1,2,3]. As a result of CGM use, the frequencies of hypoglycemia and hyperglycemia have been reduced, resulting in overall improvement in glycemia management [4,5,6,7,8,9] and improved quality of life and treatment satisfaction [10,11,12]. Two of the most popular CGM systems are the Abbott FreeStyle Libre 2 (L2) and the Dexcom G6 (G6), which both employ glucose oxidase-based amperometric technologies and are characterized by a duration of use of 14 days (L2) and 10 days (G6). While L2 comes factory-calibrated by the manufacturer, G6 is calibrated by the user after a brief run-in period. Both systems use automated wireless data transfer to a read-out unit or smartphone app [13,14].

During daily routine care, however, clinically unexplainable deviations between L2 or G6 sensor readings and blood glucose concentrations as assessed by the patient’s blood glucose meter can be observed on a regular basis [15,16]. There are multiple potential reasons for these differences. Firstly, CGMs measure glucose in the interstitial fluid (ISF), and there are known and well-characterized glucose dynamics between the ISF and blood [17,18]. In situations of rapid glucose changes, substantial differences can occur between blood and ISF glucose, which may be especially pronounced in patients with microcirculation disorders [19]. Secondly, the blood glucose meter of the patient may display inaccurate readings because of procedural patient errors, incorrectly stored test strips, outdated technology, or bias induced by interference in the test strip technologies by hematocrit or other interfering substances in the blood sample [20,21,22]. Thirdly, the factory-based calibration of L2 may not fit with the patient’s physiological condition, which may result in a consequent underestimation or overestimation of ISF glucose in comparison to capillary blood glucose readings by the patient [23]. A not-yet-well-explored fourth reason for discrepancies between ISF and blood glucose meter readings may be a potential reaction of the CGM sensors to interfering substances present in the ISF in concentrations that can substantially influence glucose readings. Such interference would explain why temporary bias between CGM and blood glucose readings can be observed and why CGM sensors sometimes prematurely cease to operate and have to be replaced [24,25]. Recently, healthcare leaders and patient advocates have requested more efforts from the manufacturers of CGM systems to investigate the impact of food components, nutritional supplements, drugs, and endogenous substances on the performance of CGM sensors [16,26]. This paper is a first step in addressing these calls to action.

The interference testing of CGM devices is not mandated by the FDA and other regulatory bodies to the same degree as is the testing of blood glucose monitoring devices, and the results are not publicly disclosed other than what is added to the label. This means that the testing performed (or not performed) is mostly left to the discretion of the manufacturers, and little information is publicly available regarding what has been tested and how it was tested [27]. Little is known about the sensitivity of the L2 and G6 systems toward other compounds besides glucose. This situation may partly be the result of a lack of in vitro screening test methods for the meaningful interference testing of CGM sensors, resulting in the requirement to conduct resource-consuming clinical studies to investigate the relevance of this problem. To support research in this area, we developed a test bench system and protocols for the dynamic in vitro performance testing of CGM sensors, providing the opportunity for cost-effective, large-scale, in vitro substance screening [28]. This method enables the search for potential interfering substances (singly or in combinations of substances) that may affect CGM sensors to a clinically relevant degree. It can also help effectively rule out substances with minimal or negligible interferent behaviors, which then may not require further investigation in clinical studies.

Here, we report the results of a comprehensive evaluation of the Abbott Libre 2 and Dexcom G6 sensors with a panel of individual food components, nutritional supplements, drugs, and endogenous substances that may be encountered by people with type 1 or type 2 diabetes. This article is a revised and expanded version of two posters that were presented at the American Diabetes Association 83rd Annual Scientific Conference in San Diego, CA, in June 2023 [29,30].

## 2. Methods

**Figure 1:**
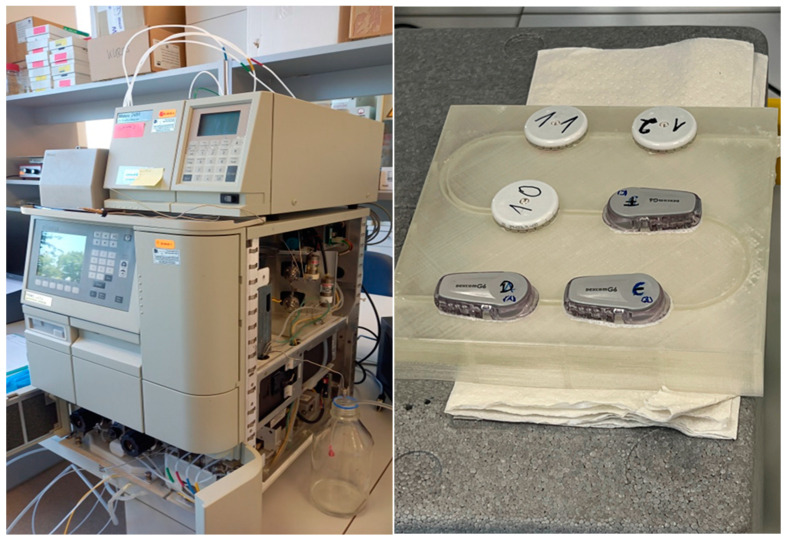
Experimental set-up of the dynamic in vitro continuous glucose monitoring sensor test bench.

We employed our standard dynamic interference testing protocol, as published previously [28], to identify substances that have the potential to influence the readings of L2 or G6 sensors. In brief, a specially designed 3D-printed test bench (PPE block: 15 cm × 15 cm × 4 cm; with an engraved macrofluidic channel: 2 mm × 10 mm × 500 mm) was equipped with three L2 and three G6 sensors. A pump, normally used to operate a high-performance liquid chromatography (HPLC) device (Waters 2695, Waters, Eschborn, Germany), was used to fill the channel with a continuous flow of phosphate-buffered saline (PBS; 18 g NaCl, 0.20 g KCl, 0.20 g KH_2_PO_4_, 1.15 g Na_2_(HPO_4_)_2_ H_2_O in 1000 mL H_2_O, pH 7.2; Carl Roth, Karlsruhe, Germany) containing 200 mg/dL glucose, at a flow rate of 1 mL/min. Additional HPLC pumps were used to supply the test bench channel with dynamically changing concentrations of candidate substances for interference testing. Samples for testing with a reference method (YSI Stat 2300 Plus, YSI, Yellow Springs, OH, USA) were taken from the end of the channel at regular time intervals during every experimental run. As temperature and oxygen partial pressure have a substantial influence on glucose oxidase-based sensor performance [31,32], all experiments were performed in a heating chamber, with a constant fluid temperature of 37 °C, and in stable oxygen partial pressure conditions. Photos of our set-up can be seen in Figure 1.

Per the standard protocol, the glucose level was maintained at 200 mg/dL to generate a baseline sensor reading, and after 30 min, the test substance (dissolved in glucose–PBS buffer) was added by a second HPLC pump. As a first step, the test substance concentration was raised dynamically, in a linear manner, to 100% of the maximally planned concentration over 60 min and was then maintained at this level for the next 30 min. In the second step, the test substance concentration was decreased linearly back to zero and maintained for another 30 min. This protocol, with increasing and decreasing substance concentrations, is effective at identifying individual substances that may interfere with the sensor signal and at eliciting the threshold level at which interference occurs [at least ±10% bias over baseline (BOB)].

For analysis, the mean of the sensor readings from the three sensors tested in parallel was calculated and compared to the baseline. The percent BOB was calculated as a measure of the level of interference of a given substance and as a benchmark parameter to allow for later comparison to other sensors.

L2 sensors are factory-calibrated for in vivo use. Factory calibration of sensors removes responsibility for calibration from the user and instead places it in the hands of the sensor manufacturer. Sensor sensitivity is determined during the sensor manufacturing process for each batch, and this information is pre-programmed into the sensor electronics such that no user interaction is required to enter the code [23]. In our in vitro system, the displayed L2 readings were 40–50% lower than the glucose concentration employed and subsequently confirmed by the reference readings [28]. With the lack of standards for CGM interference analysis, we used the Clinical and Laboratory Standards Institute guideline EP07 (Interference Testing in Clinical Chemistry, 3rd Edition), its supplement EP37 (Supplemental Tables for Interference Testing in Clinical Chemistry, 1st Edition), and the FDA definition for interference for blood glucose test strips (i.e., a deviation of > ±10% between the sensor reading and the control condition) [33,34,35] as guidelines for determining interference. If applicable, we also calculated the concentration at which a substance reached our interference definition (interference cut-off concentration, ICC). If information exists about the physiological or pharmacological concentration of the substance in the interstitial fluid after oral or parenteral administration, it may be possible to estimate the clinical relevance of the observed findings.

## 3. Results

We tested a panel of 68 individual substances, which are listed in Table 1 and Table 2, with the Abbott Libre 2 and Dexcom G6 sensors. For L2, substantial interference (BOB of at least ±10%) was seen for ten substances—ascorbic acid (BOB: +48%), dithiothreitol (+46%), galactose (>+100%), ibuprofen (+14%), icodextrin (+10%), mannose (>+100%), methyldopa (+16%), N-acetyl-cysteine (+11%), red wine (+12%), and xylose (>+100%)—while no BOB of at least ±10% was observed for the remaining substances.

With G6, we also found eleven interfering substances, four of which were the same as those found with L2: dithiothreitol (−18%), galactose (+17%), mannose (+20%), and N-acetyl-cysteine (+18%). Interference for G6 only was seen with acetaminophen (>+100%), ethyl alcohol (+12%), gentisic acid (+18%), hydroxyurea (>+100%), l-cysteine (−25%), l-Dopa (+11%), and uric acid (+33%).

Several of the substances resulted in a phenomenon consistent with sensor fouling. We observed that the G6 sensors showed a decreasing signal after exposure to a higher concentration of certain substances (e.g., dithiothreitol), such that in the follow-up period, the sensors yielded an error message and could no longer be calibrated. In consequence, once exposed to such substances, the sensors needed to be discarded and replaced for further experiments. This phenomenon was observed with dithiothreitol, gentisic acid, l-cysteine, and mesalazine. Suspected sensor fouling, in that the sensors subsequently ceased to operate after exposure to a substance, was also observed for L2 in the presence of mesalazine.

**Figure 2:**
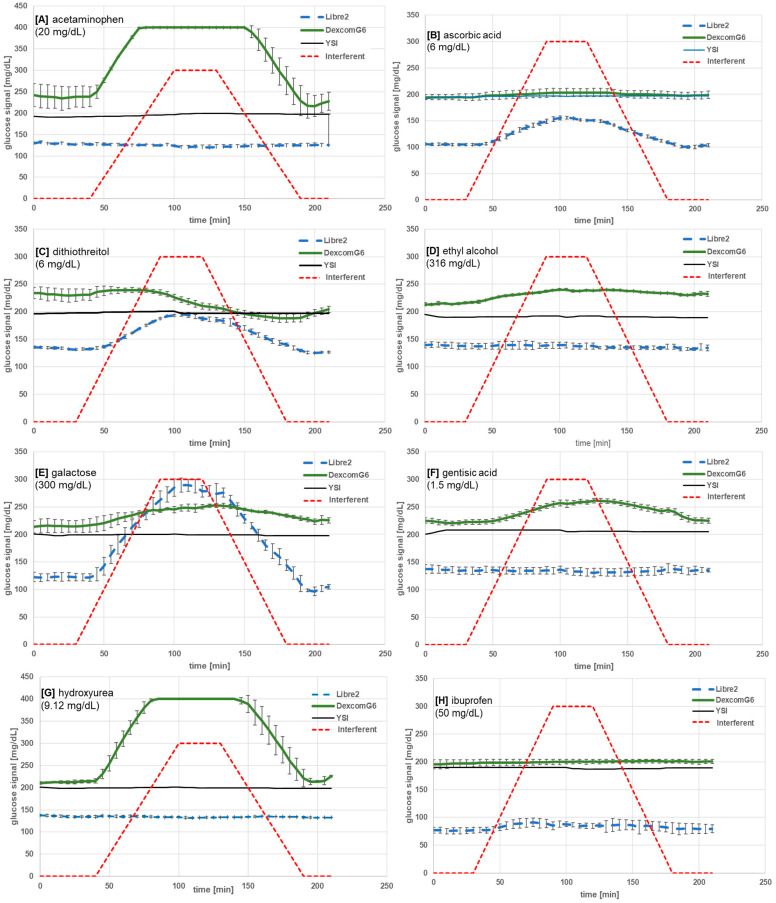

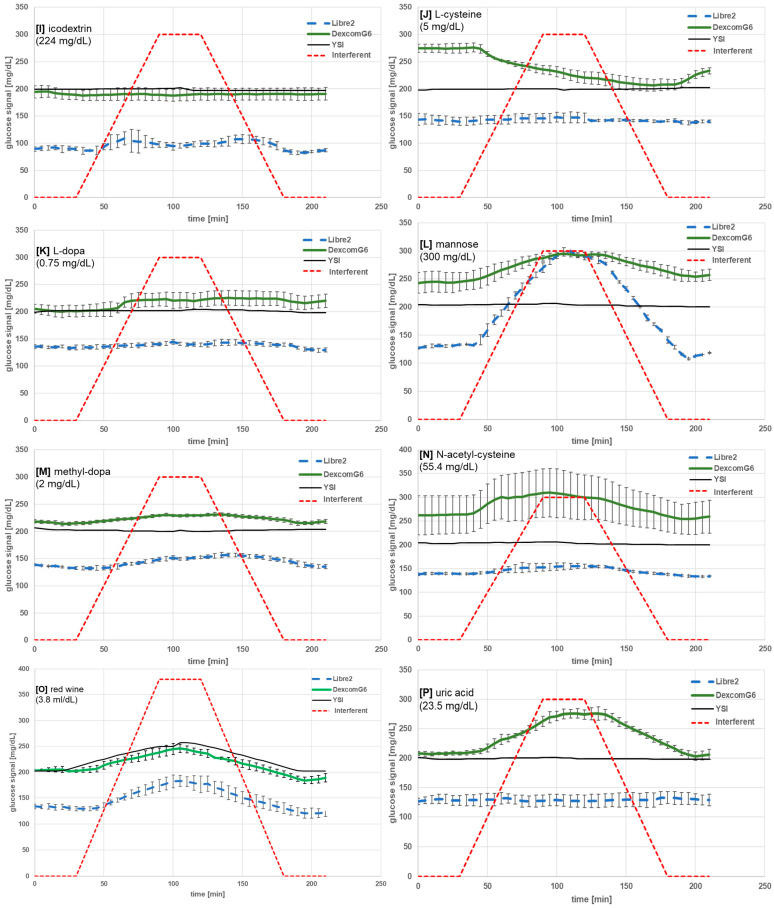

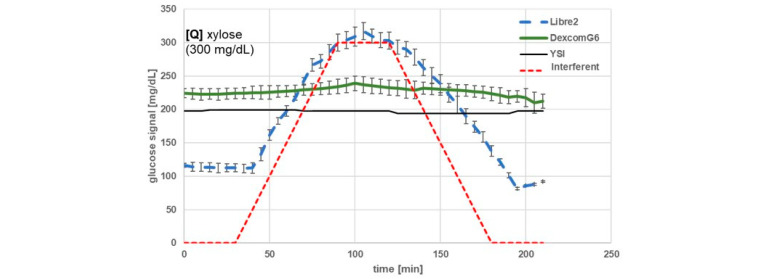
Glucose signal read-out results of the dynamic interference experiments for substances (**A**–**Q**) showing interference with either of the two sensor types. Sensors were exposed to dynamically increasing and decreasing concentrations of the substance (linear gradient) at a stable glucose concentration. The concentration displayed under the substance name indicates the highest concentration tested.

The results for each interfering substance, together with the interference cut-off concentration (ICC; threshold where at least ±10% bias over baseline first appears), are provided in Table 1. Table 2 lists the substances and tested maximal concentrations that did not show interference as determined by our BOB criteria. Graphical representations of the results obtained with the seventeen identified interfering substances are provided in Figure 2A–Q.

## 4. Discussion

Unlike existing guidelines and recommended protocols for the interference testing of blood glucose test strips and meters [36], there are no standardized procedures for the interference testing of CGM devices. For proper sensing, sensor electrodes need a quasi-stable local fluidic environment, which, while difficult to achieve in vitro, is a prerequisite to accurately test the dynamic continuous glucose measurement performance. To facilitate the detection of substance interference with CGM sensors by economic and large-scale screening, we developed and investigated a new in vitro set-up for dynamic interference testing [28]. Our experimental protocol uses a stable glucose concentration while multiple sensors are exposed simultaneously to increasing and decreasing concentrations of a potentially interfering substance. In consequence, changes in the sensor signal are not related to glucose changes but to the influence of the tested substance. As shown in Figure 2A–Q, a substance causing positive interference will lead to an increasing bias to the baseline glucose signal, which resolves once the interfering substance concentration is reduced back to zero (except in cases of sensor fouling). Although blood glucose monitoring guidelines designate an interferent as a substance that causes a greater than ±10% bias, we have adopted a threshold that includes ±10% to highlight that icodextrin causes a sufficient BOB to be of clinical concern. This consideration is especially significant if we consider the potential synergistic impact of other substances that may individually have BOBs of less than 10% but that, when present simultaneously, may similarly give rise to a BOB of clinical concern. It can also be seen in Figure 2 that the signals from Libre 2 sensors do not agree with the YSI. The commercial CGM systems that we tested have been developed and modified to yield optimal performance when used in vivo, with proprietary algorithms and designs to compensate for conditions found in the human body. While we attempted to replicate the in vivo environment as best we could (e.g., ambient temperature, oxygen partial pressure, surrogate ISF), there remained marked differences between our in vitro test rig and procedure and the normal in vivo use of these CGM systems. We were able to enter a calibration glucose value into the G6 system to have it better align with the experimental conditions, but the L2 system does not provide an opportunity to enter a calibration glucose value, and therefore, the lower signal remained. A strength of our methodology is that we first established an equilibrium condition between the sensor and the glucose flow prior to introducing the interferent flow. In this manner, we defined the magnitude of the interfering effect as the bias over baseline by comparing the deviation in the sensor reading from the zero-interferent control condition on a sensor-by-sensor basis. We recognize limitations within our test design, most notably that CGM in vitro performance can never fully imitate that seen in vivo. Furthermore, our method permitted CGM sensor reusage. Thus, some sensors would have been pre-exposed to other test substances.

One advantage of our dynamic CGM test bench methodology is the opportunity to determine a substance concentration (the ICC) that needs to be reached in the ISF to lead to a bias of at least ±10% over baseline. This may help clinicians interpret our in vitro results in light of the clinical situation. When information is available about the ISF concentration of a substance after exposure to usual amounts or doses, the potential clinical relevance of the experimental findings can be determined. An example is our result for ascorbic acid and L2, where we found the ICC for ascorbic acid on the L2 CGM to be 2.5 mg/dL. The current data on absolute bioavailability during a steady state pertain to ascorbic acid doses of 15 to 1250 mg per day, with concentrations of about 11 mg/dL in plasma being found with a daily dose of 1000 mg per day [37,38]. It is well established that ascorbic acid concentrations are much higher in tissues (and therefore in ISF) than in plasma (e.g., 24 mg/dL in the cerebrospinal fluid vs. 11 mg/dL in plasma [37]). Therefore, ascorbic acid interference is of clinical relevance, and this substance is in fact listed as an interferent in the L2 instructions for use. While the situation with ascorbic acid seems to be clear, none of the other substances that we identified as interferents with the L2 sensor in our laboratory evaluation have been identified by the manufacturer as either a known interferent or not clinically relevant.

With our in vitro protocol, we have also confirmed the previously published and clinically observed impact of hydroxyurea and acetaminophen on G6 sensor performance [39,40,41,42,43,44,45]. In our screening, we also identified nine additional substances that interfere with the G6 signal in vitro. Three of them appear to induce an irreversible change in the sensor.

In our opinion, the most important issue to address next in clinical studies is determining the concentrations of the identified in vitro interfering substances that can be expected in the ISF after regular and/or recommended substance administration. If these concentrations reach or exceed the experimentally determined ICCs, it seems likely that interference will also be observed in a clinical environment. If the dynamics of substance concentrations between blood and the ISF follow the known dynamics for glucose [17,18,19,46], then about 60% or more of the concentrations measured in the blood may be found in the ISF.

Disturbingly, there are numerous clinical scenarios where an unknown or undetected interferent affecting the sensor signal may potentially result in patient harm. For example, an interferent that causes a false elevation in the glucose reading may result in the patient undergoing unnecessary corrective treatment with insulin, which may result in hypoglycemia (e.g., after absorption of mannose and/or galactose from food). Correspondingly, glucose underestimation may result in the under-treatment of hyperglycemia. Alcohol or topical medications applied near the sensor site may alter sensor performance, resulting in false glucose fluctuations or a lack of reliable data. Electrochemical interference from drugs like N-acetyl-cysteine or high endogenous uric acid levels may reduce the reliability of glucose data, leading to a loss of confidence in the CGM device and consequent discontinuation of this beneficial technology. A sensor calibration that occurs while an interferent that causes a falsely elevated glucose reading is present may consequently lead to falsely low sensor glucose readings when the interferent is absent and subsequent prolonged periods of hyperglycemia if the user tries to achieve glucose readings on the CGM in the desired target range. Any of these situations could lead to decision-making based on inaccurate data, leading to poor outcomes.

In our opinion, it is essential to perform, and we strongly recommend, further evaluation and elucidation of the interference pattern of CGM sensors before integrating them into automated insulin delivery devices. A better understanding of sensor interference will also allow for mitigation strategies, such as selecting CGM devices based on the individual patient’s situation, teaching patients about potential interfering substances, and encouraging periodic cross-checking with fingerstick blood glucose measurements, especially when unexpected abnormal CGM readings are observed. Addressing these factors is crucial for maintaining the reliability and safety of CGM in clinical care. Furthermore, the impact of interferents needs to be considered as guidelines for using CGM devices in a hospital setting are created. The COVID-19 pandemic necessitated emergency use authorizations for CGM devices in the hospital, without the opportunity to thoroughly assess the risk/benefit ratio. This evaluation now needs to be performed. In fact, a recent consensus panel [47] felt that it was premature to fully endorse the use of CGM throughout a hospital environment (and especially in critical care) without confirmatory blood glucose testing using point-of-care devices.

## 5. Conclusions

In conclusion, employing our standardized dynamic interference testing protocol, we found several nutritional, pharmaceutical, and endogenous substances that influenced Abbott Libre 2 and Dexcom G6 signals in vitro. If confirmed by clinical trials, such interference will have to be considered when making treatment decisions while using these sensors in daily routine care.

## Figures and Tables

**Table 1:** Dynamic interference testing with Abbott Libre 2 and Dexcom G6. Results for substances interfering in vitro with both or either of the two sensors (BOB—percent bias over baseline; ICC—interference cut-off concentration = concentration where at least ±10% BOB was first reached during the experiment).

Substance	Maximum Concentration Tested (mg/dL)	BOB (%) ^1^		ICC [mg/dL]		Mean Glucose Change (SD) at 120 min vs. Baseline (mg/dL) ^2^		Type of Substance
		L2	G6	L2	G6	L2	G6	
Acetaminophen	20	−7	>+100	-	5.9	−9 (2)	Max ^3^	drug
Ascorbic acid	6	+48	+6	2.5	-	45 (1)	9 (3)	nutrient
Dithiothreitol	6	+46	−18 ^4^ fouling	3	6	52 (3)	−24 (9)	drug
Ethyl alcohol	316	+1	+12	-	300	−2 (2)	25 (2)	drug, nutrient
Galactose	300	>+100	+17	90	212	156 (2)	36 (16)	nutrient
Gentisic acid	1.5	0	+18 ^4^ fouling	-	1.0	−6 (1)	34 (4)	drug
Hydroxyurea	9.12	−3	>+100	-	0.8	−5 (0)	Max ^3^	drug
Ibuprofen	50	+14	+2	20	-	8 (2)	5 (5)	drug
Icodextrin	224	+10	−2	224	-	10 (2)	−4 (1)	drug
l-Cysteine	5	+4	−25 ^4^ fouling	-	2.5	3 (2)	−54 (18)	nutrient
l-Dopa	0.75	+7	+11	-	0.5	3 (1)	17 (21)	drug
Mannose	300	>+100	+20	77	155	163 (5)	49 (17)	nutrient
Mesalazine	0.136	0 ^3^ fouling	0 ^4^ fouling	-	-			drug
Methyldopa	2	+16	+7	2	-	15 (1)	11 (2)	drug
N-acetyl-cysteine	55.4	+11	+18	55	9.2	17 (7)	38 (21)	drug
Red wine	3.8 mL/dL	+12 ^4^	−5 ^5^	3.5	-	177 (16)	238 (6)	nutrient
Uric acid	23.5	+2	+33	-	5.9	3 (8)	66 (10)	endogenous
Xylose	300	>+100	+7	65	-	188 (9)	8 (5)	nutrient

^1^ Mean of maximum BOB. ^2^ Mean deviation in the CGM glucose reading at 120 min (the final time point at which the CGM device was exposed to the maximum interferent concentration) from baseline at the start of measurement. Standard deviation values in brackets. ^3^ Maximum CGM reading in excess of 400 mg/dL so no numerical value returned. ^4^ Sensor fouling noted post-experiment. ^5^ Additional glucose in red wine was accounted for by subtracting the differential YSI response from the CGM response when calculating the BOB.

**Table 2:** Dynamic interference testing with Abbott Libre 2 and Dexcom G6. Results for in vitro non-interfering substances (BOB: percent bias over baseline).

Substance	Maximum Concentration Tested (mg/dL)	BOB (%) ^1^		Mean Glucose Change (SD) at 120 min vs. Baseline (mg/dL) ^2^		Type of Substance
		L2	G6	L2	G6	
Alpha-tocopherol	2	+4%	+4%	+5 (2)	+7 (1)	nutrient
Amoxicillin	5	+4%	+2%	+7 (1)	+4 (0)	drug
Atenolol	0.09	+2%	+3%	+4 (1)	+6 (1)	drug
Atorvastatin	0.012	+1%	−1%	+3 (5)	−2 (2)	drug
Bilirubin conjugated	50	+1%	+7%	−9 (2)	+7 (1)	endogenous
Bilirubin unconjugated	40	+1%	+2%	−1 (5)	−3 (2)	endogenous
Budesonide	230 ng/dL	+9%	+2%	+5 (-)	+2 (1)	drug
Caffeine	5	+7%	+3%	+11 (1)	+8 (2)	nutrient
Cholesterol	500	+1%	+2%	+1 (1)	+1 (1)	endogenous
Codeine	0.015	+6%	+1%	+8 (1)	+3 (1)	drug
Creatinine	15	+2%	+1%	+2 (1)	−17 (2)	endogenous
D-fructose	18	+1%	+2%	+5 (2)	+5 (0)	nutrient
Dextromethorphan	0.0051	−4%	+2%	−3 (5)	+4 (1)	drug
Diclofenac	11.5	+2%	+2%	+1 (2)	+2 (4)	drug
Dihydrolipoate	1	+4%	+3%	+2 (2)	+4 (2)	drug
Diphenhydramine	0.011	−8%	+0%	+7 (1)	−4 (2)	drug
Dopamine	0.09	+1%	+1%	−1 (3)	+3 (1)	endogenous
Doxylamine succinate	0.01	+7%	+3%	+2 (2)	+2 (0)	drug
Dulaglutide	0.0115	−2%	+3%	+4 (2)	+7 (2)	drug
Empagliflozin	10	+1%	+1%	−8 (4)	−7 (1)	drug
Enalapril	0.01	0%	−2%	+1 (1)	−3 (0)	drug
Ephedrine	12	−3%	0%	+2 (2)	−1 (2)	drug
Gemfibrozil	6.2	0%	+1%	+4 (3)	+2 (0)	drug
Glimepiride	0.055	+5%	+3%	+3 (1)	+5 (0)	drug
Guaifenesin	0.15	+1%	−2%	0 (0)	−3 (0)	drug
Heparin	2.78	+3%	−1%	−6 (2)	−3 (2)	drug
Hydrochlorothiazide	0.05	+1%	−1%	+5 (2)	−3 (1)	drug
Insulin	500	−1%	+2%	−8 (0)	−15 (10)	endogenous, drug
Isomalt	0.09	−1%	+2%	−10 (4)	−8 (1)	nutrient
Lacitol	0.09	2%	+4%	+1 (1)	+10 (5)	sugar alcohol
Lactate	30	0%	+2%	−3 (1)	+3 (3)	endogenous
Lactose	20	−1%	+1%	−11 (2)	+1 (7)	nutrient
L-Glutathione reduced	4.6	+1%	+1%	+2 (4)	+1 (1)	endogenous
Losartan	0.075	+2%	+1%	+1 (3)	+2 (1)	drug
Mannitol	1800	+3%	−1%	+11 (1)	+2 (0)	nutrient
Malitol	0.09	+1%	+2%	−2 (0)	+3 (1)	sugar alcohol
Maltose	480	+4%	+9%	+5 (3)	+17 (22)	nutrient
Metformin	0.5	+4%	+1%	+3 (1)	+2 (0)	drug
Methylprednisolone	0.05	+6%	0%	+5 (2)	0 (4)	drug
Naproxen	7.8	−6%	−2%	−3 (2)	−8 (2)	drug
Quinidine	6.2	−5%	+2%	+1 (1)	+3 (1)	drug, nutrient
Phenylephedrine	0.065	+6%	+2%	+11 (6)	+4 (0)	drug
Reservatrol	0.08	+8%	−4%	+4 (1)	−6 (4)	drug
Rivaroxaban	0.03	+4%	+3%	+5 (2)	+7 (1)	drug
Salicylic Acid	60	+5%	+5%	+4 (1)	+9 (2)	drug
Sitagliptin	0.0233	−5%	+2%	+8 (1)	+3 (2)	drug
Sorbitol	0.09	+1%	+4%	+5 (1)	+5 (6)	sugar alcohol
Tetracycline	0.5	+4%	+2%	+4 (1)	+4 (2)	drug
Urea	24	+2%	+4%	−5 (-)	+7 (0)	endogenous
Xylitol	0.09	0%	+2%	−2 (2)	−2 (5)	sugar alcohol

^1^ Mean of maximum BOB. ^2^ Mean deviation in the CGM glucose reading at 120 min (the final time point at which the CGM device was exposed to the maximum interferent concentration) from baseline at the start of measurement. Standard deviation values in brackets.

## Data Availability

Data is on file at Lifecare Laboratory, Mainz, Germany
